# Decrease in inflammatory biomarker concentration by intervention with selenium and coenzyme Q10: a subanalysis of osteopontin, osteoprotergerin, TNFr1, TNFr2 and TWEAK

**DOI:** 10.1186/s12950-019-0210-6

**Published:** 2019-03-18

**Authors:** Urban Alehagen, Jan Alexander, Jan Aaseth, Anders Larsson

**Affiliations:** 1 Division of Cardiovascular Medicine, Department of Medical and Health Sciences, Linköping University, SE-581 85 Linköping, Sweden; 2 Norwegian Institute of Public Health, N-0403 Oslo, Norway; 3 Research Department, Innlandet Hospital Trust, Brumunddal, Norway; 4 Inland Norway University of Applied Sciences, N-2411 Elverum, Norway; 5 Department of Medical Sciences, Uppsala University, SE-751 85 Uppsala, Sweden

**Keywords:** Selenium, Coenzyme Q10, Elderly, Inflammation, Intervention

## Abstract

**Background:**

Inflammation is central to the pathogenesis of many diseases. Supplementation with selenium and coenzyme Q10 has been shown to reduce cardiovascular mortality, and increase cardiac function in elderly persons with a low intake of selenium. There are indications that one of the mechanisms of this positive effect is a decrease in inflammation.

**Methods:**

Osteopontin, osteoprotegerin, sTNF receptor 1, sTNF receptor 2 and the tumor necrosis factor-like weak inducer of apoptosis called TWEAK, were determined in plasma after 6 months and 42 months in 219 community-living elderly persons, of whom 119 received supplements of selenium (200 μg/day) and coenzyme Q10 (200 mg/day), and 101 received a placebo. Repeated measures of variance were used to evaluate the levels, and the results were validated through ANCOVA analyses with adjustments for important covariates.

**Results:**

Significantly lower concentrations of four of the five biomarkers for inflammation were observed as a result of the intervention with the supplements. Only TWEAK did not show significant differences.

**Conclusion:**

In this sub-analysis of the intervention with selenium and coenzyme Q10 or placebo in an elderly community-living population, biomarkers for inflammation were evaluated. A significantly lower concentration in four of the five biomarkers tested could be demonstrated as a result of the supplementation, indicating a robust effect on the inflammatory system. The decrease in inflammation could be one of the mechanisms behind the positive clinical results on reduced cardiovascular morbidity and mortality reported earlier as a result of the intervention. The study is small and should be regarded as hypothesis-generating, but nonetheless adds important data about mechanisms presently known to increase the risk of clinical effects such as reduced cardiovascular mortality, increased cardiac function and better health-related quality of life scoring, as previously demonstrated in the active treatment group .

**Trial registration:**

The intervention study was registered at Clinicaltrials.gov, and has the identifier NCT01443780 and registered on 09/30/2011.

## Introduction

Inflammation is a central process in the development and progress of both chronic and acute diseases. In the spectrum of cardiology, inflammation is one of the major forces in atherosclerosis [1], in acute coronary syndromes [2, 3], and heart failure [4–7]. In the aging process, signs of both increasing inflammatory activity [8], and oxidative stress are commonly observed [9].

The intake of selenium is low in Europe, and has been estimated to be around 40 μg/day [10]. The required intake of selenium in order to obtain optimal function of the intracellular enzyme glutathione peroxidase, and the extracellular protector selenoprotein P has been discussed, and Xia et al. reported a needed intake of 75 μg/day of selenium for adult Caucasians [11]. Moreover, there is a need for increased intake of selenium during conditions of increased oxidative stress and inflammation [12]. This is also applicable to persons living in regions with otherwise optimal selenium content of the soil, as in the US. There are reports indicating increased cardiovascular disease and mortality due to a low intake of selenium. Salonen et al. reported a 2.9 fold increase in cardiovascular mortality in those with a low selenium intake [13]. Our group have also reported increased cardiovascular mortality associated with low intake of selenium in healthy, elderly community-living persons [14].

Coenzyme Q_10_ (ubiquinone) is present in all living cells in the body, where it acts as an important antioxidant, but it is also active in the mitochondrial respiratory chain. The endogenous production of coenzyme Q_10_ declines after the age of 20, and in the myocardial cells it is reduced to about half at the age of 80 years [15]. There is an important interrelationship between selenium and coenzyme Q_10_, as the cytosolic selenoenzyme thioreductase is needed for the reduction of ubiquinone to ubiquinol, which is the active antioxidant form of coenzyme Q_10_ [16, 17].

Our group have previously reported effects by intervention with selenium and coenzyme Q10 on two biomarkers for inflammation; sP-selectin and C-reactive protein, with significantly fewer signs of inflammation in those receiving active treatment, as compared with those in the placebo group [18]. As an inhibitory effect on the inflammatory activity caused by supplementation with selenium and coenzyme Q10 would be important, we wanted to validate the effects reported earlier on sP-selectin and a high sensitivity assay of CRP (hsCRP). We evaluated the impact on five other biomarkers for inflammation; osteopontin, osteoprotegerin, soluble tumor necrosis factor receptor 1 (TNFr1), soluble tumor necrosis factor receptor 2 (TNFr2), and the tumor necrosis factor-like weak inducer of apoptosis, called TWEAK.

The aim of this sub-study was to investigate a possible influence of supplementation with selenium and coenzyme Q10 for 3.5 years on the inflammatory process, with emphasis on cardiovascular aspects, in an elderly Swedish population, using five different biomarkers of inflammation as indicators of the inflammatory activity.

## Methods

### Subjects

This is a secondary analysis of a subgroup of 219 individuals from a prospective randomized double-blind placebo-controlled trial in an elderly community population of 443 individuals with an age range of 70–88 years. We were able to report significantly reduced cardiovascular mortality, increased cardiovascular function, and less concentration of the cardiac peptide NT-proBNP as a response to less myocardial wall tension as a result of the intervention. The trial has been previously reported [19, 20]. The participants in the main study received the intervention for 48 months, and were re-examined every 6 months. At inclusion, new patient records were obtained, all participants went through a clinical examination, the New York Heart Association functional class (NYHA class) was assessed, and an ECG and Doppler echocardiographical examinations were performed with the participant in the left lateral position. The ejection fraction (EF) readings were categorized into four classes, with interclass limits placed at 30, 40 and 50% [21, 22]. Normal systolic function was defined as EF ≥ 50%, while severely impaired systolic function was defined as EF *<* 30%.

Informed consent was obtained from each patient. In the main study, 221 individuals received active supplementation of 200 μg/day organic selenium (SelenoPrecise®, Pharma Nord, Denmark), plus 200 mg/day of coenzyme Q_10_ (Bio-Quinon®, Pharma Nord, Denmark), and 222 individuals received a placebo.

The present subgroup comprised 219 participants (118 individuals on active treatment and 101 on placebo), and the figures were based on the number of participants still living and within the study having delivered blood samples both after six and after 42 months.

### Ethical approval and consent to participate

The study was approved by the Regional Ethical Committee and conforms to the ethical guidelines of the 1975 Declaration of Helsinki. (The Medical Product Agency declined to review the study protocol since the study was not considered a trial of a medication for a certain disease but rather one of food supplement commodities that are commercially available). This study was registered at Clinicaltrials.gov, and has the identifier NCT01443780. Since it was not mandatory to register at the time of the study start, the study has been registered retrospectively.

### Biochemical analyses

Blood samples were collected after six and 42 months while the participants were resting in a supine position. Pre-chilled, EDTA vials containing plasma were used. The vials were centrifuged at 3000 *g*, + 4 °C, and were then frozen at − 70 °C. No sample was thawed more than once.

### Determination of the inflammatory biomarkers

Plasma Osteopontin, Osteoprotegerin, TNFr1, TNFr2 and TWEAK/ TNFSF12 were analyzed using commercial ELISA kits (DY1433, DY805, DY321B, DY726 and DY1090, R&D Systems, Minneapolis, MN, USA). The assays had a total coefficient of variation of approximately 6%. Those performing the measurements were blinded as to the purpose of the study and without knowledge of the clinical data.

### Statistical methods

Descriptive data are presented as percentages or mean ± SD. A Student’s unpaired two-sided T-test was used for continuous variables and the chi-square test was used for analysis of one discrete variable. Repeated measures of variance were used in order to obtain better information on the individual changes in the concentration of the biomarker analyzed, compared to group mean values.

As the analysis of variance (ANOVA) algorithm can handle a slight non-Gaussian distribution, non-transformed data were applied in the repeated measures of variance evaluation. In the analysis of covariance (ANCOVA) evaluation, both transformed and non-transformed data were applied, with no significant difference in the results.

In the ANCOVA evaluation, the biomarker concentration after 42 months was used as an independent variable. In the model, adjustments were made for age, smoking, hypertension, ischemic heart disease (IHD), biomarker concentration after 6 months, hs-CRP, and supplementation with selenium and coenzyme Q10.

*P*-values < 0.05 were considered significant, based on a two-sided evaluation. All data were analyzed using standard software (Statistica v. 13.2, Dell Inc., Tulsa, OK).

## Results

The study population in this sub-study consisted of 219 participants, of which 118 received active treatment, and 101 received placebo. In the population, 17% had diabetes, 70% had hypertension and 17% had IHD (Table 1; submitted as separate file due to table size). It could also be seen that 13% had treatment with ACE inhibitors, and 77% had treatment with beta-blockers.

**Table 1:** Baseline characteristics of the study population receiving intervention of a dietary supplementation of selenium and coenzyme Q10 combined during 4 years

	Active	Placebo	p-value
N	118	101	
Age years mean (SD)	76.2 (3.1)	76.3 (3.1)	0.74
Males/Females n	58/60	43/58	
History			
Diabetes n (%)	20 (16.9)	18 (17.8)	0.87
Hypertension n (%)	81 (68.5)	72 (71.3)	0.67
IHD n (%)	22 (18.6)	16 (15.8)	0.59
NYHA class I n (%)	71 (60.2)	58 (57.4)	0.68
NYHA class II n (%)	29 (24.6)	30 (29.7)	0.39
NYHA class III n (%)	18 (15.3)	12 (11.9)	0.47
NYHA class IV n (%)	0	0	
Medications			
Anticoagulants n (%)	9 (7.6)	9 (8.9)	0.73
ACEI n (%)	15 (12.7)	14 (13.9)	0.80
ARB n (%)	4 (3.4)	7 (6.9)	0.23
Beta blockers n (%)	44 (37.3)	33 (32.7)	0.48
Digitalis n (%)	5 (4.2)	1 (0.9)	0.14
Diuretics n (%)	39 (33.1)	33 (32.7)	0.95
Statins n (%)	27 (22.9)	17 (16.8)	0.27
Examinations			
EF < 40% n (%)	7 (5.9)	4 (4.0)	0.51
Atrial fibrillation n (%)	6 (5.1)	7 (6.9)	0.56
Lab			
Hb < 120 g/L (%)	11 (9.3)	9 (8.9)	0.92
hsCRP g/L (SD)	2.60 (2.60)	5.60 (22.15)	0.34

Note: *ACEI* ACE- inhibitors, *ARB* Angiotension receptor blockers, *EF* Ejection fraction, *hsCRP* C-reactive protein analysed with high sensitivity assay, *IHD* Ischemic heart disease, *IQR* Inter quartile range, *NYHA* New York Heart Association functional class, *SD* Standard Deviation

From the evaluations at inclusion, no difference could be seen between the two groups as based on the group mean evaluation and thus the two populations were considered balanced (Table 1).

### Osteopontin and intervention with selenium and coenzyme Q10

At the start of the sub-study, that is after 6 months, there was no significant difference in osteopontin concentration between the active treatment group, and the placebo group (65,913 mmol/L vs. 65,395 mmol/L; T = 0.1; *P* = 0.92). However, after 42 months a highly significant difference between the two groups could be found (60,067 mmol/L vs. 71,632 mmol/L; T = 3.72; *P* = 0.0003) with a lower concentration in the active treatment group.

Analyzing the placebo group, a borderline significant increase in the concentration of osteopontin could be seen between six months and 42 months (65,395 mmol/L vs. 71,633 mmol/L; T = 1.90; *P* = 0.059); however, in the active group no significant change in concentration could be found (65,913 mmol/L vs. 60,067 mmol/L; T = 1.17; *P* = 0.24).

**Fig. 1:**
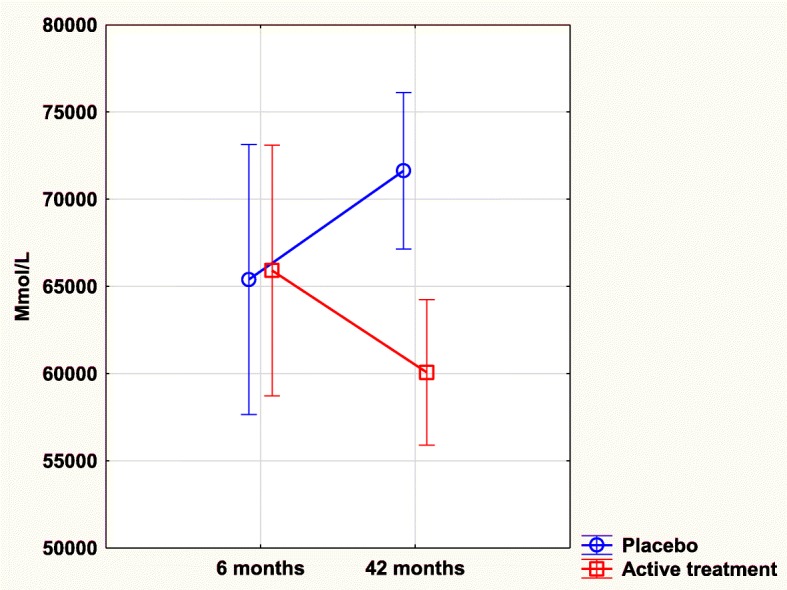
Concentration of Osteopontin after 6 and 42 months in the placebo and selenium and coenzyme Q10 treatment groups in the total study population. Note: Current effect: F(1, 216) = 4.5913, *p* = .03325. Note: Evaluation performed by use of repeated measures of variance methodology. Note: Blue curve: Placebo; Red curve: Active treatment group. Values ±95% C, Vertical bars denote 0.95 confidence intervals

Applying repeated measures of variance, a highly significant difference in concentration of osteopontin between the placebo group and the active group could be seen (F = 4.59; *P* = 0.03) with a lower concentration in the active treatment group (Fig. 1).

Validating the results through an ANCOVA evaluation, a significant difference in osteopontin concentration could be seen between those receiving active treatment and those receiving placebo, with a lower concentration in the active treatment group (*P* = 0.004).

### Osteoprotegerin and intervention with selenium and coenzyme Q10

After 6 months, there was no significant difference in osteoprotegerin concentration between the active treatment group, and the placebo group (5931 mmol/L vs. 5725 mmol/L; T = 0.5; *P* = 0.61). However, after 42 months a highly significant difference between the two groups could be found (5877 mmol/L vs. 6552 mmol/L; T = 2.73; *P* = 0.007) with a lower concentration in the active treatment group.

Validating the above results of osteoprotegerin concentration difference, an ANCOVA evaluation was performed, where the biomarker displayed a significantly lower concentration in the active treatment group compared to the placebo group, and this was also found after adjustments of important covariables (*P* = 0.04).

**Fig. 2:**
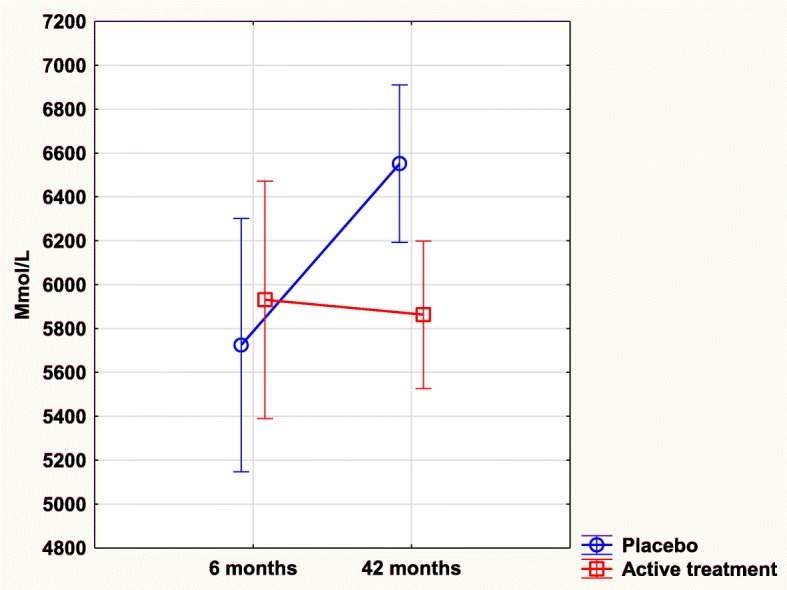
Concentration of Osteoprotegerin after 6 and 42 months in the placebo and selenium and coenzyme Q10 treatment groups in the total study population. Note: Current effect: F(1, 214) = 4.6059, *p* = .03299. Note: Evaluation performed by use of repeated measures of variance methodology. Note: Blue curve: Placebo; Red curve: Active treatment group. Values ±95% CI. Vertical bars denote 0.95 confidence intervals

Repeated measures of variance evaluation also demonstrated a significant difference in osteoprotegerin with a lower concentration in the active treatment group (F = 4.61; *P* = 0.03) (Fig. 2).

### TNFr1 and intervention with selenium and coenzyme Q10

No significant difference in TNFr1 concentration could be seen between the active treatment group, and the placebo group after 6 months of intervention (2873 mmol/L vs. 2903 mmol/L; T = 0.2; *P* = 0.81). After 42 months, a highly significant difference between the two groups was found with a higher concentration in the placebo group (3012 mmol/L vs. 2656 mmol/L; T = 2.83; *P* = 0.005).

In the placebo group we found no significant change in the concentration at 6 months in comparison with that at 42 months (2902 mmol/L vs. 3012 mmol/L; T = 0.78; *P* = 0.44). However, in the active treatment group, a significant reduction of concentration could be found during the same period (2873 mmol/L vs. 2656 mmol/L; T = 1.98; *P* = 0.049).

**Fig. 3:**
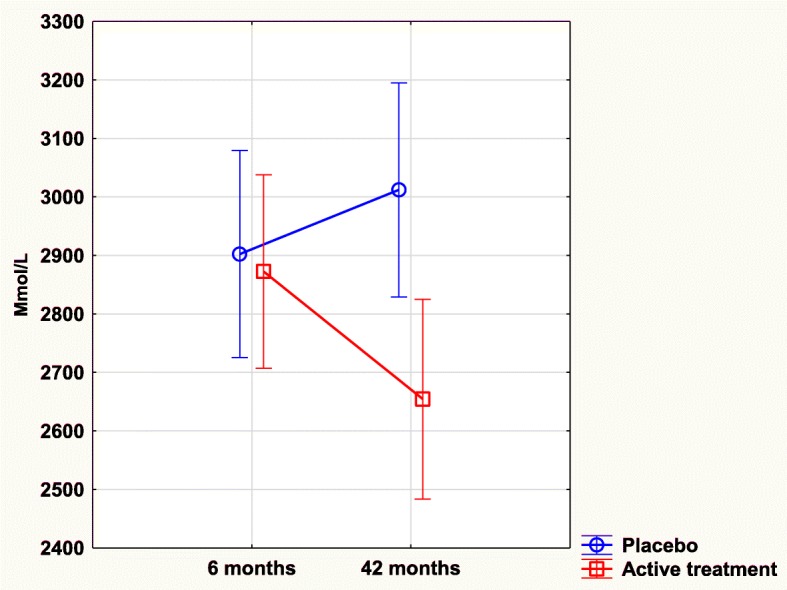
Concentration of TNFr1 after 6 and 42 months in the placebo and selenium and coenzyme Q10 treatment groups in the total study population. Note: Evaluation performed by use of repeated measures of variance methodology. Note: Current effect: F(1, 215) = 4.8064, *p* = .02943. Note: Blue curve: Placebo; Red curve: Active treatment group. Values ±95% CI. Vertical bars denote 0.95 confidence intervals

By applying repeated measures of variance, a significant difference in concentrations could be demonstrated (F = 4.81; *P* = 0.03) with a lower concentration in the active treatment group (Fig. 3).

### TNFr2 and intervention with selenium and coenzyme Q10

No significant difference in concentrations of TNFr2 could be seen between the active treatment and the placebo groups after 6 months of intervention (5621 mmol/L vs. 5569 mmol/L; T = 0.21; *P* = 0.84). However, after 42 months of intervention a slightly lowered level of concentration of TNFr2 could be seen in the active treatment group as compared to the placebo group (5398 mmol/L vs. 5984 mmol/L; T = 1.95; *P* = 0.05).

Validating the result by applying the ANCOVA model, the significant effect of intervention with selenium and coenzyme Q10 on TNFr2 after 42 months remained even after adjustments with several important covariates (F = 4.49; *P* = 0.04), that is, there was a lower concentration of the biomarker in the active treatment group.

**Fig. 4:**
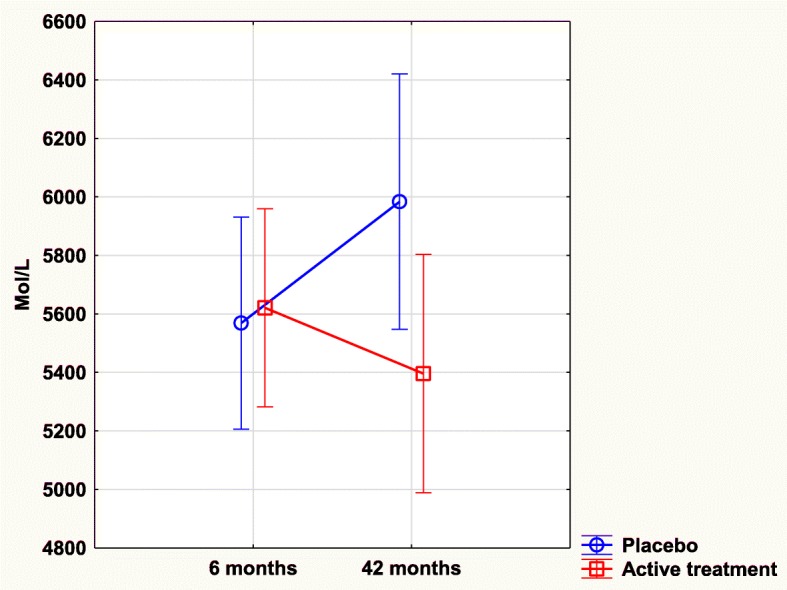
Concentration of TNFr2 after 6 and 42 months in the placebo and selenium and coenzyme Q10 treatment groups in the total study population. Note: Evaluation performed by use of repeated measures of variance methodology. Note: Current effect: F(1, 215) = 5.6269, *p* = .01857. Note: Blue curve: Placebo; Red curve: Active treatment group. Values ±95% CI. Vertical bars denote 0.95 confidence intervals

Applying repeated measures of variance methodology, a highly significant difference in concentration of TNFr2 between the active treatment group and the placebo group could be found (F = 5.63; *P* = 0.02) with a lower concentration in the active treatment group (Fig. 4).

### TWEAK and intervention with selenium and coenzyme Q10

After 6 months, no difference in concentration of TWEAK could be found between the active treatment group and the placebo group (3820 mmol/L vs. 4250 mmol/L; T = 0.25; *P* = 0.80). After 42 months of intervention, still no difference between the two groups could be seen (4014 mmol/L vs. 4959 mmol/L; T = 0.53; *P* = 0.60). Moreover, no difference be demonstrated by applying repeated measures of variance (F = 0.14; *P* = 0.71).

## Discussion

In this study, we conducted further examination of the impact of supplementation with selenium and coenzyme Q10 on inflammation using five different biomarkers. In many cardiovascular diseases, inflammation is one of the most important components of the disease; thus, a possible positive influence of the disease through reduced inflammation would also have an impact on the need for health resources of society.

The two bone biomarkers osteopontin and osteoprotegerin are of special interest as they are also associated with inflammation.

Osteopontin levels are significantly associated with the presence and the extent of cardiovascular disease independently of traditional risk factors [23]. Also, osteopontin levels are higher in patients presenting signs of vascular calcification than those without and correlate with the number of segments affected. An increased concentration of osteoprotegerin has been consistently associated with the incidence and prevalence of coronary artery disease [24].

We have previously reported positive effects on two biomarkers of inflammation; sP-selectin, and hsCRP [18]. However, as both biomarkers may also reflect processes other than inflammation, we wanted to validate the result by applying five other biomarkers of inflammation.

Although the two related biomarkers, osteopontin and osteoprotegerin, are involved in the bone metabolism, it is well known that they also reflect inflammation, and they can therefore be used as biomarkers for inflammatory activity.

Kim et al. demonstrated increased apoptosis of endothelial progenitor cells as a result of an increased level of osteoprotegerin, and they used free radical scavengers to negate the effect [25]. The present results show that intervention with selenium and coenzyme Q10 reduced levels of osteoprotegerin in line with the reduced cardiovascular mortality. A previously reported association between the level of osteopontin and cardiovascular events for those with carotid artery stenosis further strengthens our reasoning [26].

sTNFr1 and r2 are both reported to play an active part in inflammation [27, 28]. There is also an association between the concentration of both receptors, and frailty in elderly persons. This association with frailty is stronger than that with age, which could be interpreted to mean that for the individual, the inflammatory activity is important as regards overall health [29]. This concurs with the results from our study, where intervention with selenium and coenzyme Q10 showed effects both on clinical endpoints and on biomarkers for oxidative stress [20], inflammation [18] and microRNA [30].

Regarding the biomarker TWEAK, the literature reports not only associations between the concentration of the biomarker and inflammation of the skin [31], but also an inflammatory-related up-regulation of endothelial cells accompanying increased levels of TWEAK [32]. Therefore, we expected that the intervention with selenium and coenzyme Q10 would reduce the concentration of TWEAK; however, no significant change occurred. The reason for this is presently unclear. It has been reported, however, that using a plasma concentration of TWEAK, as we have done, gives a significantly lower concentration of TWEAK, as compared to a serum concentration of TWEAK [33]. However, this insufficient reliability should affect both treatment groups.

When interpreting the signs of decreased inflammation as a result of the intervention, an attractive explanation is the fact that the present ageing population has a suboptimal intake of selenium, and a suboptimal endogenous production of active coenzyme Q10 (ubiquinol). As three of the most important selenoenzymes (glutathione peroxidase, thioredoxin reductase, and selenoprotein P) are important in the anti-oxidative defense, it is reasonable that a normalization of the deficiencies of the two essential substances would lead to improved global health and reduced inflammatory activity.

Our group has previously reported signs of reduced oxidative stress supporting the mechanisms discussed above [20] . Our previous observations of changed expression of microRNA as a result of the intervention [30] are also compatible with the hypothesis that normalization of the ubiquinol and selenium status in the studied group has a protective potential as regards inflammation and cardiovascular health. From the patient perspective, we have also reported improved health-related quality of life, and more days outside hospital, as a result of the intervention [34]. Also, a reduced cardiovascular mortality after 10 and after 12 years as a result of four years of intervention of selenium and coenzyme Q10 has also been presented [35, 36]. In order to evaluate a possible association between the level of inflammation biomarker and mortality, the cardiovascular mortality after 12 years have been evaluated in the 1st versus the 4th quartiles of concentration of each biomarker (Table 2).

**Table 2:** Relation between cardiovascular mortality after 12 years of follow-up, and the 1st and 4th quartiles of concentration of the five biomarkers for inflammation evaluated

Variable	CV mortality Q1	CV mortality Q4	Χ^2^-value	p-value
Osteopontin, n (%)	19 (17.4)	36 (33.0)	7.03	0.008
Osteoprotegerin, n (%)	20 (18.5)	35 (33.0)	5.89	0.015
sTNFr1, n (%)	17 (15.7)	37 (34.6)	10.14	0.002
sTNFr2, n (%)	23 (21.3)	32 (29.6)	1.98	0.16
TWEAK, n (%)	30 (27.8)	35 (32.4)	0.55	0.46

Note: Q1: 1st quartile; Q4: 4th quartile

From the obtained results in the present sub-study, it is clear that intervention with selenium and coenzyme Q10 gives significantly decreased inflammation in four out of five biomarkers of inflammation evaluated, when compared with placebo. This could be one of the mechanisms explaining the apparent protection against cardiovascular events reported previously as a result of the intervention [19].

### Limitations

The studied population was of limited size, 219 individuals; therefore, the reported results should be interpreted with caution. However, as the differences between the active treatment and placebo groups were highly significant, it is probable that the results reflect real changes. The report should be regarded as hypothesis-generating, and as such it has interesting information that should be subjected to further research.

The study population was not sampled primarily, but chosen because they lived in the same rural community and were in the same age stratum. The results could therefore be subject to bias because of a lower threshold to participate among those with known or unknown disease, but with symptoms, hoping for a diagnosis or treatment adjustment, leading to a greater preponderance of those individuals. This situation could theoretically result in an even higher level of inflammation compared to other healthy populations of corresponding age. However, as the main study population was randomized into two groups, it could be expected that those given active treatment would have a similar health situation as those on placebo, as was also seen from the balanced covariates in the baseline characteristics.

Furthermore, the study population represented a specific age stratum. It is therefore difficult to extrapolate the obtained information into other age groups.

Finally, the study population was an ethnically homogenous Caucasian population; thus, we do not know if the information could be applied to other populations.

## Conclusion

Here we report on the impact following intervention with selenium and coenzyme Q10 on inflammation as measured by the concentrations of the five biomarkers: osteopontin, osteoprotegerin, sTNFr1, sTNFr2 and TWEAK. In all, except for TWEAK, the intervention caused a significant reduction in the biomarker concentrations. These results strengthen the previous results reported for sP-selectin and C-reactive protein, which also showed the same signs of reduced inflammatory activity.

The positive impact on inflammation could be part of the mechanism explaining the positive clinical results reported earlier. However, as the sample size was limited, the results should be interpreted with caution.

## Abbreviations

ANCOVA: Analysis of covariance
ANOVA: Analysis of variance
EF: Ejection fraction
hsCRP: high sensitivity assay of CRP
IHD: Ischemic heart disease
NYHA class: New York Heart Association functional class
TNFr1: soluble tumor necrosis factor receptor 1
TNFr2: soluble tumor necrosis factor receptor 2
TNF-α: Tumor necrosis factor-α
TWEAK: the tumor necrosis factor like weak inducer of apoptosis, called TWEAK
